# Controlling of two destructive zooplanktonic predators in *Chlorella* mass culture with surfactants

**DOI:** 10.1186/s13068-021-01873-6

**Published:** 2021-01-14

**Authors:** Xiaobin Wen, Aoqi Zhang, Xiaoyan Zhu, Lin Liang, Yan Huo, Kaixuan Wang, Youzhi Yu, Yahong Geng, Yi Ding, Yeguang Li

**Affiliations:** 1 Key Laboratory of Plant Germplasm Enhancement and Specialty Agriculture, Wuhan Botanical Garden, Chinese Academy of Sciences, Wuhan, 430074 China; 2 Center of Economic Botany, Core Botanical Gardens, Chinese Academy of Sciences, Wuhan, 430074 China; 3 University of Chinese Academy of Sciences, Beijing, 100049 China

**Keywords:** *Chlorella*, Predator, Flagellates, Ciliates, Bio-contamination, Surfactant

## Abstract

**Background:**

Predatory flagellates and ciliates are two common bio-contaminants which frequently cause biomass losses in *Chlorella* mass culture. Efficient and targeted ways are required to control these contaminations in *Chlorella* mass cultivation aiming for biofuel production especially.

**Results:**

Five surfactants were tested for its ability to control bio-contaminations in *Chlorella* culture. All five surfactants were able to eliminate the contaminants at a proper concentration. Particularly the minimal effective concentrations of sodium dodecyl benzene sulfonate (SDBS) to completely eliminate *Poterioochromonas* sp. and *Hemiurosomoida* sp. were 8 and 10 mg L^−1^, respectively, yet the photosynthesis and viability of *Chlorella* was not significantly affected. These results were further validated in *Chlorella* mass cultures in 5, 20, and 200 m^2^ raceway ponds.

**Conclusions:**

A chemical method using 10 mg L^−1^ SDBS as pesticide to control predatory flagellate or ciliate contamination in *Chlorella* mass culture was proposed. The method helps for a sustained microalgae biomass production and utilization, especially for biofuel production.

## Background

*Chlorella* is a genus of unicellular green microalgae that has long been used as a model organism to study photosynthesis [1]. *Chlorella* biomass is rich in protein, vitamins, and minerals. The success of *Chlorella* mass culture during the late 1940s created a stable *Chlorella* industry, primarily for human nutrition and animal feed [2, 3]. Recently, *Chlorella* is considered a candidate for bioenergy and bioremediation owing to its ability to grow fast, uptake nutrients in wastewaters, and synthesize a large amount of TAGs or carbohydrates in cells [4, 5].

However, the current autotrophic technologies that are used for mass production of *Chlorella* biomass are facing challenges from biological contamination. Biological contamination occurs frequently in *Chlorella* mass culture in the widely used cultivation systems including circular and raceway ponds [3, 6–8]. Zooplanktonic predators, such as ciliates, rotifers, amoeba, and flagellates, are the most common contaminants as reported in the literature [8, 9]. According to surveys by Ma et al. [10, 11], contamination by the predatory flagellate *Poterioochromonas malhamensis* in *Chlorella* culture can occur at any time throughout the year at any growing location. Ciliates are also widely spread and can cause serious problems to microalgae cultivation under broad climate conditions [12]. Invasion by these predators usually develops into bio-contamination. The most direct effect of such contamination is reduction of biomass yield. For example, the cell density of *Chlorella* has been shown to decrease from 4.0 × 10^8^ to 1.0 × 10^8^ cells mL^−1^ within three days, whereas that of the grazer *P. malhamensis* increased to 1.1 × 10^6^ cells mL^−1^ from 1.0 × 10^3^ cells mL^−1^ [11]. Moreno-Garrido and Canavate [12] reported that grazing ciliates can visually clarify dense outdoor mass cultures of *Dunaliella salina* within 2 days. Similarly, over 60% of *Chlorella* biomass can be digested in a short time due to the explosive growth of predators such as flagellates or ciliates, according to the authors’ experiences. Thus, the control of biological contamination is very important for the mass production of *Chlorella* in open systems.

Biological contaminations are different in their occurrence, development, and contamination mechanisms [6]. Many contaminations have occurred in an associative or sequential manner and interacted with the target microalgae [13]. These factors make the control of biological contamination very complicated. Methods have been suggested to overcome the challenges of biological contamination, such as filtration, changes of the environmental conditions such as medium pH, and use of chemical additives including quinine, formaldehyde, ammonia, and hydrogen peroxide [6, 8, 10, 12]. These methods are helpful in controlling different types of zooplanktonic contaminants. However, methods such as filtration and changes of medium pH are inefficient to apply in large scale, and some chemical additives, for example, ammonia and ammonium bicarbonate, are not applicable in microalgal cultivation where nitrogen limitation is necessary to induce TAG or astaxanthin accumulation since the addition of such chemicals will relieve nitrogen deficiency. Thus, more efficient and targeted ways are still required. Wang et al. [6] suggested that strain selection (non-susceptibility/resistance to biological pollutants) is the most practicable approach to cope with biological invasions, yet it is very time-consuming because a single algal species is unlikely to excel in all the required characteristics, such as resistance to biological pollutants, rapid growth, high product content, wide tolerance of environmental conditions, and other qualities that facilitate industrial production.

Surfactants (or ‘surface active agents’) are organic compounds that can modify the solution properties both within the bulk of the solution and at the solid/water interface [14], and they have been recognized as having certain cytotoxicity [15–17]. Cell membranes are the primary target for the toxicological effects of surfactants on cells, which are known to be loss of cell viability and cell lysis [18, 19]. Here, we report on a simple and efficient chemical method, using surfactant as a single additive, to control the contamination of predatory flagellates and ciliates in *Chlorella* mass culture. Flagellates and ciliates, specifically *Poterioochromonas* sp. and *Hemiurosomoida* sp., have several similarities in the context of contamination in *Chlorella* mass culture in the present study. They are both unicellular, and can swim and graze on *Chlorella* cells and especially lack a resistant structure outside the plasma membrane [20–22] in comparison to *Chlorella*. These characteristics create possibilities for the targeted control of *Poterioochromonas* and *Hemiurosomoida* without inhibition on *Chlorella* growth. Five widely used surfactants in household cleaning products are used as pesticides and their effects on the two predators and *Chlorella* are investigated and compared. The application of this method is also discussed and recommended based on field testing.

## Results

### Toxic effects of surfactants on predator growth and reproduction

The successive transfer cultures of the two predators (*Poterioochromonas* sp. and *Hemiurosomoida* sp.) were established first as described in Methods. Using these successive transfer cultures, the efficacies of the five selected surfactants for controlling *Poterioochromonas* sp. and *Hemiurosomoida* sp. were evaluated. Toxic effects on both *Poterioochromonas* sp. and *Hemiurosomoida* sp. were observed for all five surfactants, namely sodium dodecyl benzene sulfonate (SDBS), coconut diethanolamide (CDEA), sodium dodecyl sulfate (SDS), fatty alcohol polyoxyethylene ether (AEO-7), and alcohol ethoxysulphate (AES).

**Fig. 1:**
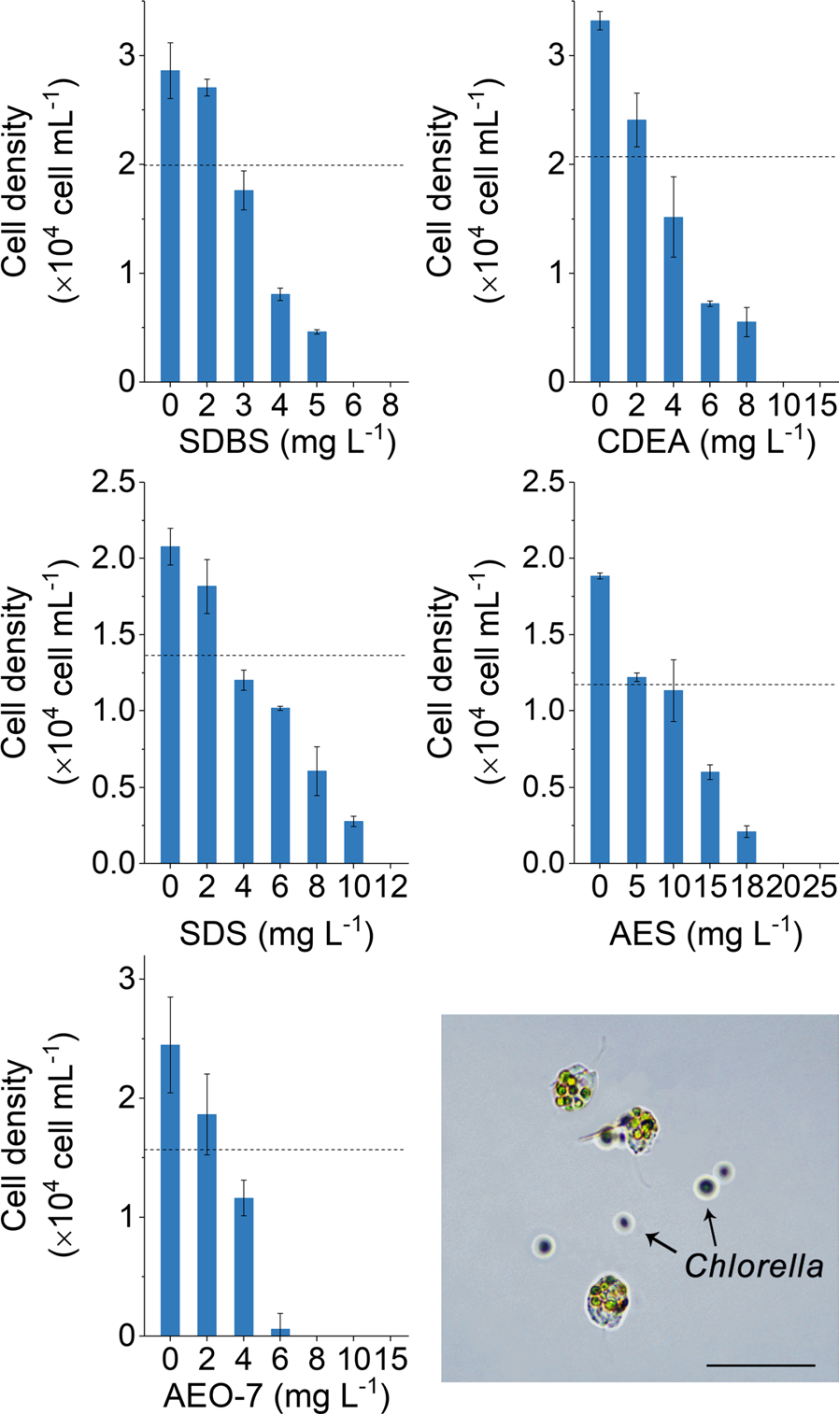
Toxic effects of five surfactants on *Poterioochromonas* sp. growth. Dash lines represent cell densities at the beginning of the experiment. Columns indicate cell densities at 24 h after surfactant addition. Error bars represent the standard deviations of 3 replicates. Microscopic image of *Poterioochromonas* sp. with *Chlorella* cells inside their body is shown at the bottom-right corner. Scale bar = 30 µm

As shown in Fig. 1, a greater than 30% increase in cell densities of *Poterioochromonas* sp. were obtained after 24 h cultivation without surfactant addition, demonstrating the viability of the *Poterioochromonas* sp. cultures. However, the cell densities decreased in the cultures supplemented with any one of the five surfactants. For example, the cell density of the living *Poterioochromonas* sp. was 2.8 × 10^4^ cells mL^−1^ in the culture without SDBS addition, yet it decreased to 1.8 × 10^4^ cells mL^−1^ in the culture supplemented with 3 mg L^−1^ SDBS and further decreased to less than 100 cells mL^−1^ with 6 mg L^−1^ SDBS treatment. No living *Poterioochromonas* sp. were observed microscopically when the SDBS concentration was further increased to 8 mg L^−1^, which we considered as the complete control of *Poterioochromonas* sp. contamination.

The decreasing trend in *Poterioochromonas* sp. density with increasing surfactant concentration was found for all five tested surfactants, suggesting that they affect the predator *Poterioochromonas* sp. similarly. However, for each surfactant, the minimal effective concentrations to completely control the contamination were different. SDBS and AEO-7 were the most powerful reagents, eliminating *Poterioochromonas* sp. completely at concentrations not lower than 8 mg L^−1^. Second, the efficacies of CDEA and SDS on *Poterioochromonas* sp. were similar and their minimal effective concentrations were 10 and 12 mg L^−1^, respectively. AES showed weak efficacy on controlling of *Poterioochromonas* sp., with a minimal effective concentration of 20 mg L^−1^.

**Fig. 2:**
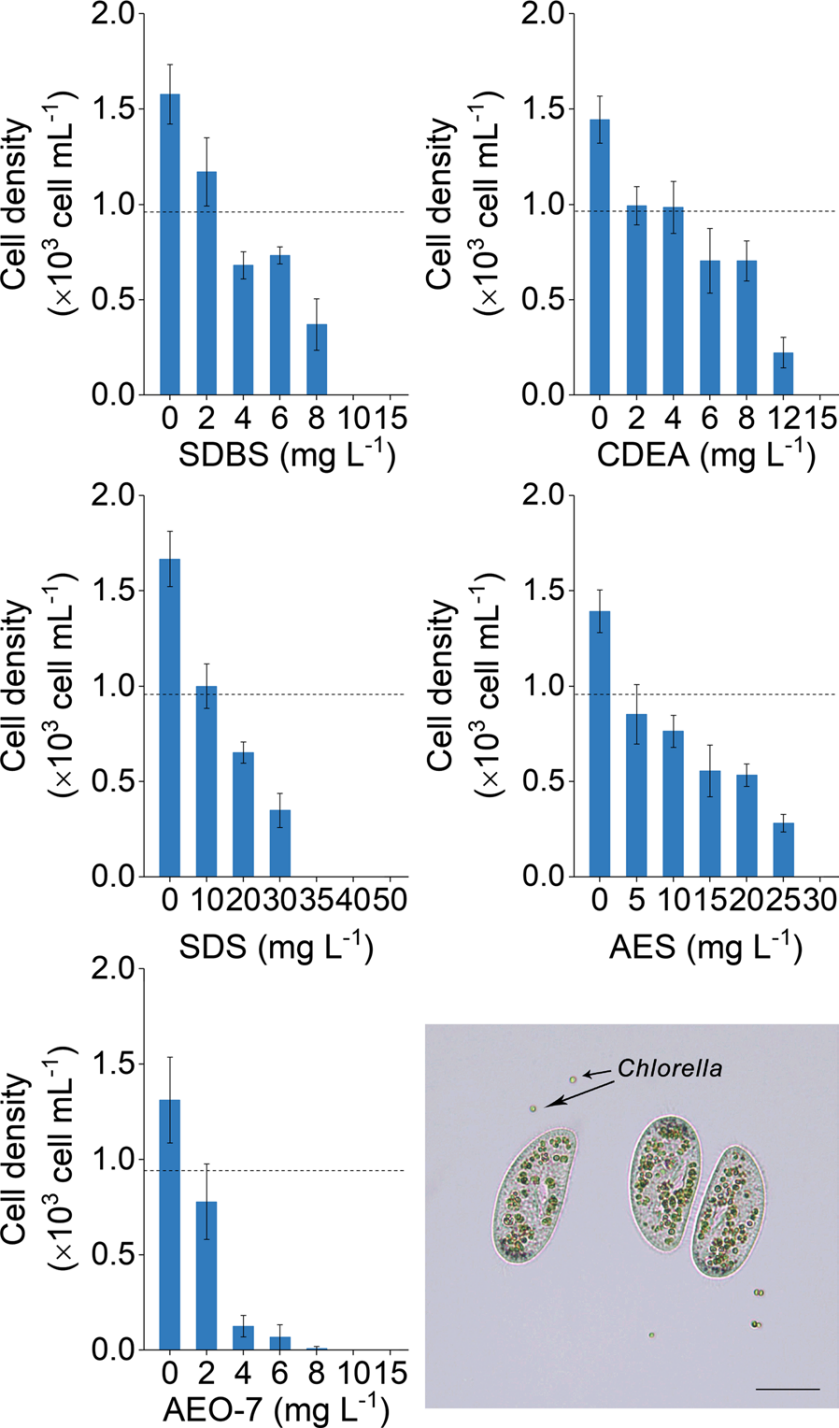
Toxic effects of five surfactants on *Hemiurosomoida* sp. growth. Dash lines represent cell densities at the beginning of the experiment. Columns indicate cell densities at 24 h after surfactant addition. Error bars represent the standard deviations of 3 replicates. Microscopic image of *Hemiurosomoida* sp. with *Chlorella* cells inside their body is shown at the bottom-right corner. Scale bar = 60 µm

Toxic effects of the five surfactants on *Hemiurosomoida* sp. were also observed (Fig. 2). The viability of *Hemiurosomoida* sp. was shown by an increased in cell densities, which were more than 40% higher in comparison to the initial density in the culture without surfactant supplementation. *Hemiurosomoida* sp. densities decreased significantly after surfactants addition. Taking the SDBS treatment as an example, almost 60% decrease in the *Hemiurosomoida* sp. density, from 1.6 × 10^3^ to 680 cells mL^−1^, was obtained when 4 mg L^−1^ SDBS was supplemented into the culture. A further increase in the SDBS concentration (10 mg L^−1^) led to the complete elimination of *Hemiurosomoida* sp. and no living cells were observed under the microscope. The general trends of decreasing cell densities with increasing surfactant concentrations were also detected for the five surfactants. However, the efficacies against *Hemiurosomoida* sp. were not the same as that for *Poterioochromonas* sp. The most powerful one was AEO-7, which eliminated *Hemiurosomoida* sp. at a concentration of 8 mg L^−1^. The next ones were SDBS and CDEA, the minimal effective concentrations of which were 10 and 15 mg L^−1^, respectively. The complete elimination of *Hemiurosomoida* sp. by AES was only obtained at 30 mg L^−1^. A substantial difference was observed in SDS, which had a minimal effective concentration of 12 mg L^−1^ for *Poterioochromonas* sp., but at least 35 mg L^−1^ SDS was needed to completely eliminate *Hemiurosomoida* sp.

### Effects of the five surfactants on *Chlorella* growth

**Fig. 3:**
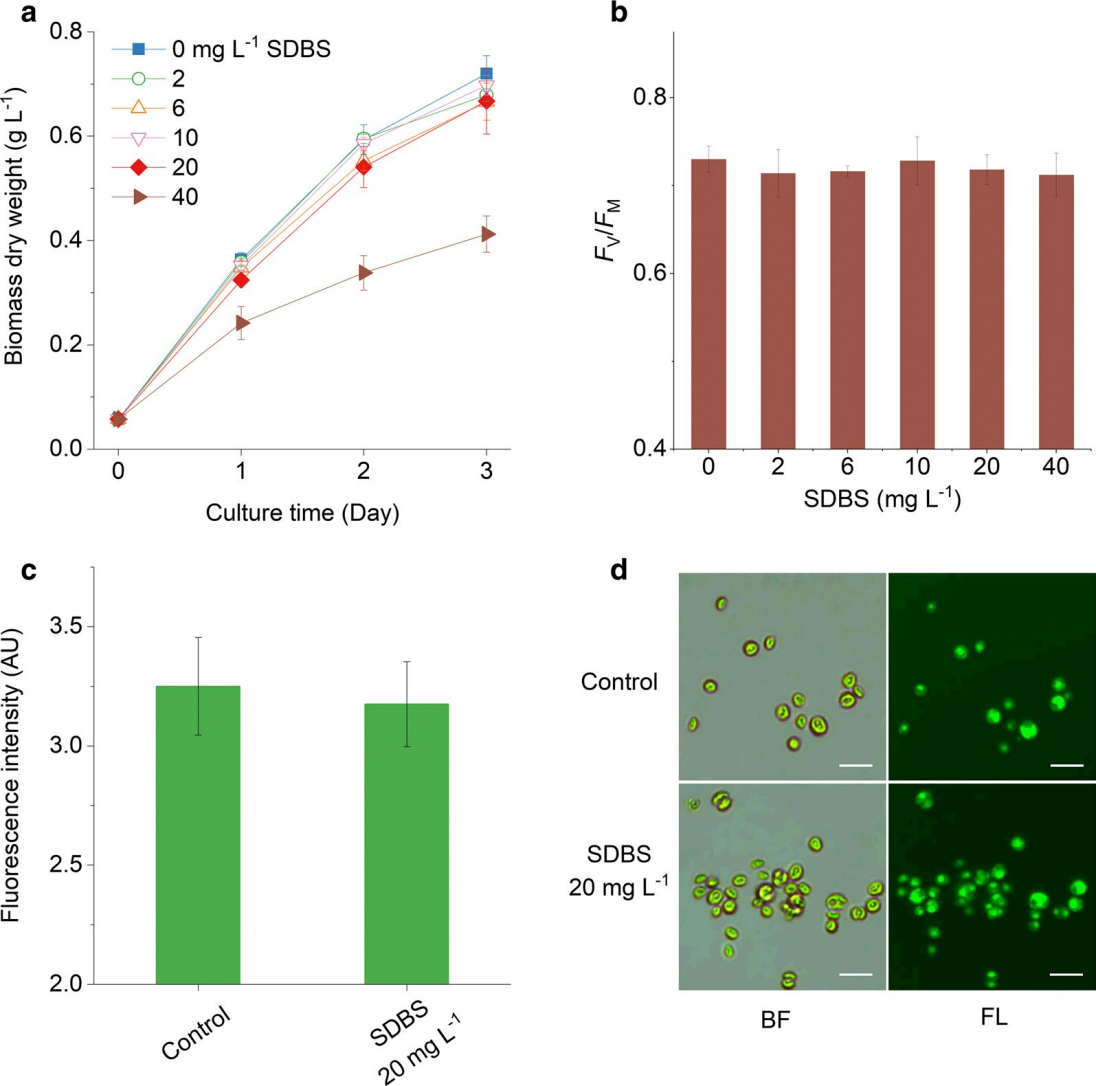
Effects of sodium dodecyl benzene sulfonate (SDBS) on *Chlorella pyrenoidosa* XQ-20044. **a** Cell growth, **b** photosynthetic activity, **c** FDA staining fluorescence intensities, and **d** bright field (BF) and fluorescence (FL) image of the cells. Scale bars = 10 µm. Error bars represent the standard deviations of 3 replicates

*Chlorella pyrenoidosa* XQ-20044 was cultured under different concentrations of the five surfactants to evaluate the surfactant effects on cell growth, photosynthetic activity, and viability. Data of the SDBS exposure experiment are shown in Fig. 3 as an example; other data concerning CDEA, SDS, AES, and AEO-7 are provided in Additional file 1.

The time courses of the *Chlorella* biomass DW showed no significant difference (*P* > 0.05) when the SDBS concentration was less than 20 mg L^−1^ (Fig. 3a). The biomass DW of the culture having no SDBS supplementation reached 0.72 g L^−1^ on day 3, with an average growth rate of 0.84 d^−1^. Smaller but insignificant (*P* > 0.05) biomass DW (0.67 g L^−1^) and growth rate (0.82 d^−1^) were obtained in the culture with 20 mg L^−1^ SDBS supplementation. However, the biomass DW was only 0.41 g L^−1^ with a significantly decreased (*P* < 0.05) growth rate of 0.66 d^−1^ when the SDBS concentration was further increased to 40 mg L^−1^.

The photosynthetic activity of *Chlorella* (Fig. 3b) showed that in comparison to the SDBS-free culture, the changes in the photochemical yield of *Chlorella* cells were very small after 3 days of exposure to 20 mg L^−1^ SDBS. The ratio between variable fluorescence and maximum fluorescence (*F*_V_/*F*_M_) of *Chlorella* was 0.72 in the SDBS-treated (20 mg L^−1^) culture in the present study. This value fell into the general *F*_V_/*F*_M_ range of dark-adapted green microalgae, suggesting that the photosynthetic activity of *C. pyrenoidosa* XQ-20044 was not influenced by SDBS at concentrations lower than 20 mg L^−1^.

FDA staining (Fig. 3c, d) clearly showed membrane integrity and viability of the *Chlorella* cells, with similar fluorescein fluorescence intensities in both the SDBS-treated (20 mg L^−1^) and the contrast culture. All the above results suggested that *Chlorella* biomass yield may be reduced due to over exposure to SDBS, but the influences of SDBS was negligible at a concentration not higher than 20 mg L^−1^.

### Application of sodium dodecyl benzene sulfonate (SDBS) as a pesticide to control flagellates and ciliates grazing on *Chlorella* in raceway pond

**Fig. 4:**
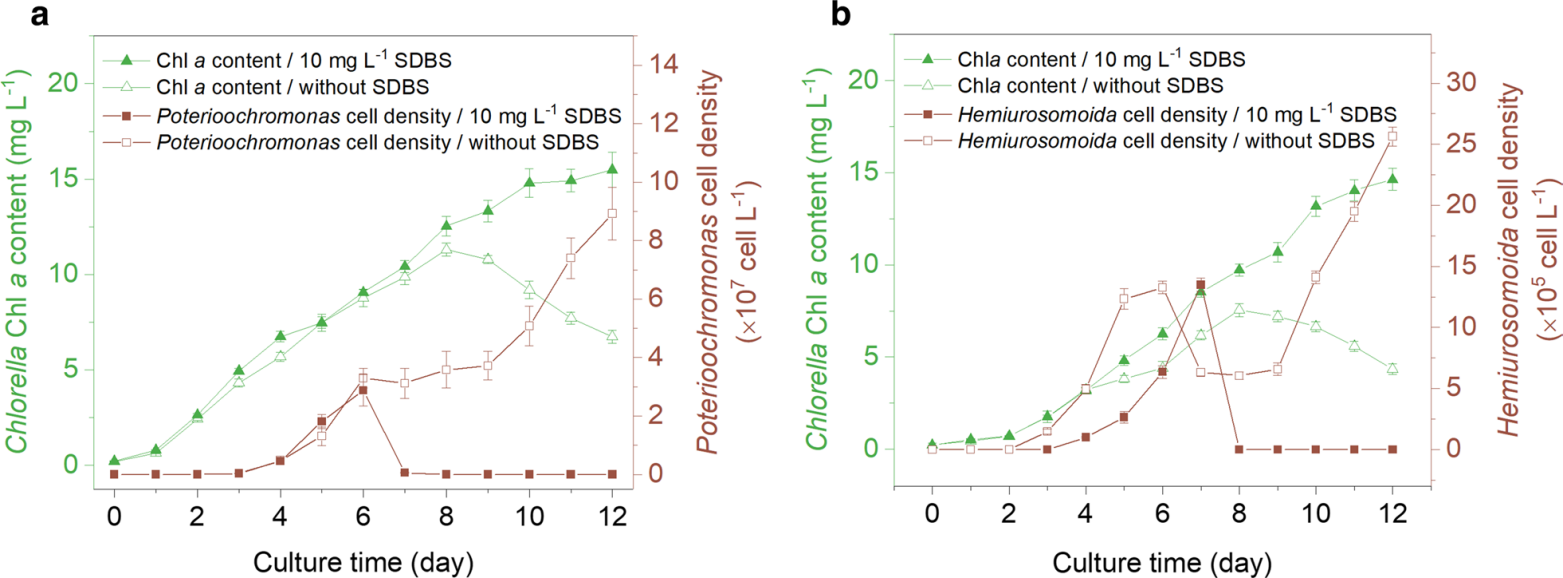
Changes in cell density of *Chlorella* and inoculated contaminating organisms in 5-m^2^ raceway ponds. The contaminating organisms were *Poterioochromonas* sp. (**a**) and *Hemiurosomoida* sp. (**b**), with open symbols indicating the cultivation without SDBS treatment, and closed symbols indicating the cultivation treated with 10 mg L^−1^ SDBS on the 6th day (**a**) and the 7th day (**b**). Error bars represent the standard deviations of 3 replicate measurements

The above results demonstrate that SDBS and AEO-7 were powerful surfactants for controlling *Poterioochromonas* and *Hemiurosomoida* contaminants while had little effect on *Chlorella* growth. Considering that SDBS is more easily degraded in natural environment and is less toxic to aquatic organisms than AEO-7 [23], SDBS was further tested outdoors to validate the laboratory data. According to our observation, naturally occurring contaminations of *Poterioochromonas* sp. or *Hemiurosomoida* sp. can be observed generally on days 2–4 of a newly inoculated *Chlorella* culture in an outdoor raceway pond (unpublished results). This trend was successfully mimicked by the addition of *Poterioochromonas* sp. or *Hemiurosomoida* sp. “seeds” into the *Chlorella* culture ponds (Fig. 4). 18S rDNA-based metagenomic data for identification of the contaminating species can be seen in Additional file 2. The predator densities increased continuously for 3–4 days after inoculation. For example, *Hemiurosomoida* sp. increased from 1.0 × 10^5^ cells L^−1^ on the 4th day to 1.4 × 10^6^ cells L^−1^ on the 7th day. At this time the cultures were treated with 10 mg L^−1^ SDBS, and contaminations in other parallel cultures were not treated and allowed to develop.

As shown in Fig. 4, cell densities of the predators *Poterioochromonas* sp. and *Hemiurosomoida* sp. increased consistently for 3 or 4 days. The target microalgae *C. pyrenoidosa* XQ-20044 also showed a quick increase in cell density (indicated by Chl *a* content) during this period because the predator populations were not large enough to have a significant grazing effect on *Chlorella*. The increase in predator densities continued thereafter in the cultivations without SDBS addition. When the densities of *Poterioochromonas* sp. and *Hemiurosomoida* sp. reached approximately 3.6 × 10^7^ and 6.4 × 10^5^ cells L^−1^, respectively, the *Chlorella* density decreased due to predation. By comparison, almost all the *Poterioochromonas* sp. and *Hemiurosomoida* sp. cells disintegrated and disappeared in one day in the cultivations after SDBS addition (10 mg L^−1^) on the 6th day and 7th day, respectively, with the *Chlorella* growth kept as normal.

**Fig. 5:**
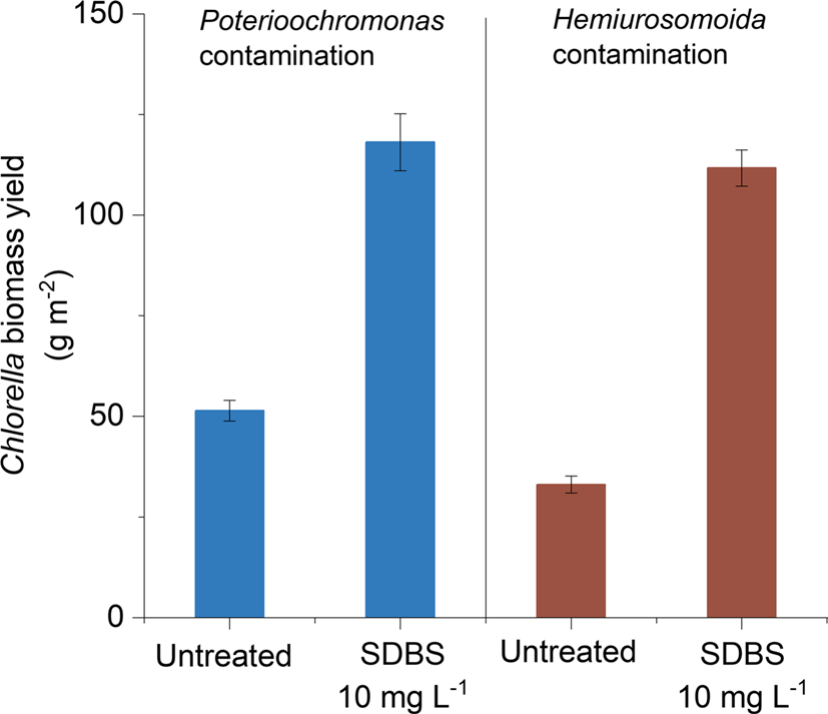
Comparison of biomass yields between the contaminated cultivations with and without SDBS treatment. Error bars represent the standard deviations of 3 replicate measurements

Overall, the final *Chlorella* biomass concentration reached 0.6 g L^−1^ after a 12-day cultivation applying SDBS as pesticide. It was only 0.26 g L^−1^ if the *Poterioochromonas* contamination was not controlled and 0.17 g L^−1^ if the *Hemiurosomoida* contamination was not controlled (Fig. 5). These data suggest that by applying 10 mg L^−1^ SDBS as a pesticide to control *Poterioochromonas* sp. or *Hemiurosomoida* sp. contamination, the reduction in *Chlorella* biomass yield, which was estimated to be greater than 60% owing to predation, can be avoided. Actually, economic loss caused by biological contamination was much bigger than expected because the residual *Chlorella* biomass could only be used as low-quality raw materials when no effective steps were taken to manage the contaminations. The working concentration of SDBS (10 mg L^−1^) was slightly higher than the minimal effective concentration to eliminate *Poterioochromonas* sp. in the laboratory. This was to simplify the application that using one uniform concentration to control both *Poterioochromonas* and *Hemiurosomoida* contaminations.

**Fig. 6:**
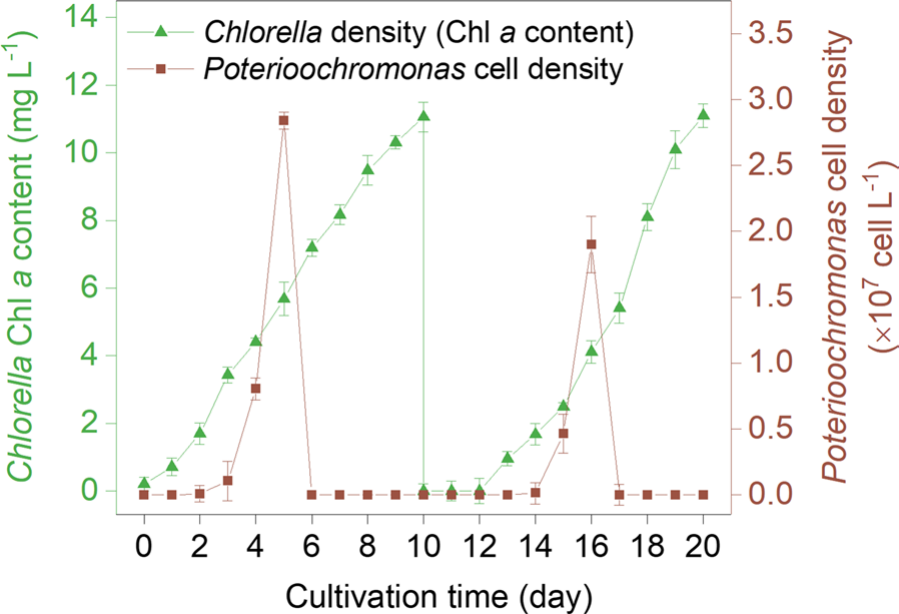
Cascade cultivations of *Chlorella* in raceway ponds and contamination control using SDBS. The first 10 days of cultivation was conducted in a 20-m^2^ pond and the next 10 days was in a 200-m^2^ raceway pond. Both observed contaminants were *Poterioochromonas* sp. and 10 mg L^−1^ SDBS was added on the 5th and 16th day. Error bars represent the standard deviations of 3 replicate measurements

SDBS pesticide was also applied in 20 and 200 m^2^ cascade cultures of *Chlorella* at October 2019 (Fig. 6). The cascade cultures were initiated in a 20-m^2^ raceway pond. After 10 days of cultivation the culture suspension was used as seed for a larger culture in a 200-m^2^ raceway pond. Two rounds of contamination naturally occurred during the process, both of which were *Poterioochromonas* sp. contaminations. The first round of *Poterioochromonas* contamination was observed early on the 2nd day in the 20-m^2^ pond. The cell density of *Poterioochromonas* increased gradually from 7.6 × 10^4^ to 1.1 × 10^6^ cells L^−1^ on the 3rd day, and then drastically increased with densities on the 4th and 5th days reaching 8.1 × 10^6^ and 2.8 × 10^7^ cells L^−1^, respectively. During this time, the *Chlorella* density was not significantly influenced because the predator density was relatively low. SDBS addition (10 mg L^−1^) on the 5th day resulted in a sharp decrease in *Poterioochromonas* sp. density and the predator was not observed over the following days. The algal biomass increased continuously after addition of the SDBS pesticide. A cell density of 11.1 mg Chl *a* L^−1^ (0.42 g DW L^−1^, alternatively 8.4 g m^−2^ d^−1^) was observed on the 10th day. The biomass yield of *Chlorella* was comparable to those previously reported [24, 25]. On the 10th day, the culture was scaled up into a 200-m^2^ raceway pond and 4 days later the second round of *Poterioochromonas* contamination was observed. Development of the second round of contamination was very similar to the previous one observed in the 20-m^2^ pond. SDBS pesticide (10 mg L^−1^) successfully eliminated *Poterioochromonas* sp. once again, without damaging *Chlorella* growth.

## Discussion

### Surfactants as novel pesticide for controlling biological contamination in *Chlorella* culture

In the present study surfactants were used as pesticide to control ciliate and flagellate contaminations in *Chlorella* culture. Among the selected five surfactants, SDBS, SDS, and AEO-7 met the basic requirements of a pesticide for the control of *Poterioochromonas* sp. and *Hemiurosomoida* sp. in *Chlorella* culture. First, the complete control (elimination) of the two predators could be achieved by the addition of any one of the tested surfactants at a proper concentration (Figs. 1 and 2). Second, the surfactants SDBS, SDS, and AEO-7, which eliminated the two predators at the minimal effective concentrations, had little effects on *Chlorella* growth. Particularly, the minimal effective concentrations of SDBS for the complete elimination of *Poterioochromonas* sp. and *Hemiurosomoida* sp. were as low as 8 and 10 mg L^−1^, respectively. However, SDBS concentrations as high as 20 mg L^−1^ had no effect on photosynthetic activity, cell membrane integrity, and biomass accumulation of *C. pyrenoidosa* XQ-20044 (Fig. 3).

### Possible mechanisms of SDBS pesticide for controlling bio-contaminations

Previous studies regarding the aquatic toxicity of anionic surfactants [15, 16, 26, 27] showed that green algae were more tolerant to anionic surfactant (such as SDBS) exposure compared to invertebrates including daphnia, ciliates, flagellates, and bacteria. These results were consistent with those of the present study. Such differential tolerance between *Chlorella* and the two predators provide evidence that these surfactants can be used as pesticides to control contamination in *Chlorella* mass cultures.

One remaining question is why did the surfactants only eliminate predators such as *Poterioochromonas* sp. and *Hemiurosomoida* sp. rather than *Chlorella*? Microscopical observation at 24 h after the addition of surfactants showed that the predators decreased in numbers or even disappeared from the *Chlorella* culture. In fact, these changes occurred in less than 10 min after the addition of surfactants. Continuous microscopic monitoring revealed that the predator cells, whether it was *Poterioochromonas* sp. or *Hemiurosomoida* sp., disintegrated shortly once the SDBS concentration got close to the minimal effective concentration (Additional file 3). However, the free-living *Chlorella* cells that were not swallowed retained their morphological and physiological integrity (Fig. 3).

As mentioned before, cell membranes are the primary target for the toxicological effects of surfactants on cell [18, 19]. *Poterioochromonas* and *Hemiurosomoida* are unicellular organisms that lack a rigid or resistant structure (composed of insoluble non-hydrolysable biopolymers) outside the plasma membrane [20–22]. These cells were so sensitive that the lipid bilayers were disrupted immediately when enough surfactants were available in the medium.

One of the cell structures that differs *Chlorella* from the two predators (*Poterioochromonas* sp. and *Hemiurosomoida* sp.) is its cell wall. Numerous species of green microalgae including *C. pyrenoidosa* have a two-layer cell wall with a classical polysaccharidic layer that is proximal to the cytoplasmic membrane and a thin outer layer [28, 29]. The outer layers are often trilaminar organized (termed as the trilaminar sheath, TLS) and are composed of insoluble non-hydrolysable biopolymers exhibiting an unusually high resistance to non-oxidative chemical degradation [30, 31]. In a study concerning bio-toxicity of environmental chemicals, Gwenael Corre et al. [28] found that the presence of a TLS in *C. emersonii* was associated with a very high resistance to anionic (DBS) and nonionic (TX-100) detergents at all growth stages. We consider this is also the reason why the photosynthetic capacity and viability of *C. pyrenoidosa* were not significantly affected by 20 mg L^−1^ SDBS in the present study. The TLS of *C. pyrenoidosa* may have worked as a protective structure against SDBS.

### Applications of SDBS pesticide in *Chlorella* mass culture

SDBS is one of the most commonly used anionic surfactants for cleaning application [32], and it is easy to manufacture, store, transport, and handle [32], which facilitate its application in microalgae production. The current price of SDBS is $1.4 per kilogram in China. In other words, material cost is only 1.4 U.S. dollar for applying SDBS pesticide to a 100 m^3^ *Chlorella* cultivation. It is very cheap in comparison to other methods [33].

SDBS is generally regarded as a biodegradable surfactant and its degradation rate may be as high as 97–99% under aerobic conditions [34]. We harvested the SDBS-treated *Chlorella* in other study [33] to find if there was residual SDBS in the harvested *Chlorella* biomass, and no SDBS was detected. Thus, the environmental impact of this method is slight. However, more investigations are still needed. Our previous studies have also shown that the surfactants such as SDBS are unable to induce changes in algal lipid synthesis [35, 36]. Therefore, it will be very cheap, convenient, and safe to use SDBS as a new pesticide in microalgal mass cultivation, especially for biofuel production. Avoiding target biomass reduction is a necessary principle for biological contamination control in microalgal cultivation. Predator reproduction and *Chlorella* biomass loss are both becoming faster and bigger with the extension of time for a contaminated *Chlorella* cultivation. So, early detection and treatment are crucial for minimizing algal biomass reduction. From this point of view, 10 mg L^−1^ SDBS should be added as soon as predators are observed microscopically to prevent further reproduction of predators. Since cell densities of the predator *Poterioochromonas* and *Hemiurosomoida* are relatively low at this time, 10 mg L^−1^ SDBS would be adequate for completely eliminating the contaminants.

The tolerable SDBS concentration for *C. pyrenoidosa* is 20 mg L^−1^, which is at least two times that of the minimal effective concentration for eliminating the predators *Poterioochromonas* sp. and *Hemiurosomoida* sp. Such a difference is very helpful for outdoor application. Even the SDBS pesticide is required once again in a short time, the *Chlorella* will not be affected negatively. On the other hand, predator cells are disintegrated in a short time after SDBS addition, thus protective time of the pesticide will depend on how fast SDBS is degraded. Further studies should concern degradation dynamics of SDBS in microalgal culture and how environmental factors affect the degradation process.

## Conclusions

All five selected surfactants were effective for eliminating *Poterioochromonas* sp. and *Hemiurosomoida* sp. contamination in the laboratory. Further studies indicated SDBS (10 mg L^−1^) is an efficient pesticide to control the contaminations without damaging *Chlorella*. One of the principles for SDBS pesticide application is early detection and treatment of contaminations. The surfactant SDBS directly acts on the unprotected plasma membrane of the predators; therefore, the efficacy of SDBS as a pesticide may be universal. The authors expect a broad spectrum of anti-biocontaminations to be developed using the method outlined in the present study.

## Methods

### Chemicals used for the control of biological contaminants

Five surfactants, specifically sodium dodecyl benzene sulfonate (SDBS, CAS NO. 25155-30-0), coconut diethanolamide (CDEA, CAS NO. 68603-42-9), sodium dodecyl sulfate (SDS, CAS NO. 151-21-3), fatty alcohol polyoxyethylene ether (AEO-7, C12, CAS NO. 68131-39-5), and alcohol ethoxysulphate (AES, C12, CAS NO. 68891-38-3), were tested for their effects on microalgae and biological contaminants.

### *Chlorella* strain and test for its growth under surfactants exposure

*Chlorella pyrenoidosa* XQ-20044 was used in the present study. It was provided by the Algae Culture Collection of Wuhan Botanical Garden, Chinese Academy of Sciences. The algal seed were grown autotrophically in shaking flasks under light intensity of 50 μmol m^−2^ s^−1^ for 14 h per day and constant temperature (25 °C).

To study the effects of the SDBS surfactant on *Chlorella* growth, the seed cultures were inoculated into a bubbled column photobioreactor (PBR) at an initial density of OD_540_ = 0.3 (approximately 0.05 g L^−1^ dry weight). Light intensity at the surface of the column PBR was set at 200 μmol m^−2^ s^−1^ with a light/dark cycle of 14/10 h. A thermostatic water bath was used to maintain constant culture temperature of 30 °C. The culture suspension was agitated with 200 mL min^−1^ air enriched with 1% CO_2_ (v/v). The basal growth medium was BG-11. Concentrated SDBS solutions were added to the culture columns immediately after inoculation to reach final SDBS concentrations of 0, 2, 6, 10, 20, and 40 mg L^−1^. Each cultivation was run in triplicate and proceeded for 3 days. The biomass dry weight (DW) and photosynthesis activity were monitored every day during cultivation. Fluorescein diacetate (FDA) dye was used to indicate the cell viability of *Chlorella pyrenoidosa* XQ-20044. Effects of the other four surfactants were evaluated by comparing DWs of the culture under the same conditions.

### Predator isolation and successive transfer cultures

The predators, namely *Poterioochromonas* sp. and *Hemiurosomoida* sp., were frequently observed and caused biomass loss during our test cultivations of *C. pyrenoidosa* XQ-20044 in open raceway ponds from 2011 to 2019. We isolated *Poterioochromonas* sp. and *Hemiurosomoida* sp. cells and fed them *C. pyrenoidosa* XQ-20044 cells. After repeatedly re-isolation and feeding, steady cultures of the two predators were established. For maintenance, the cultures were kept at room temperature (25 °C) with manual shaking (twice per day). Diluted *C. pyrenoidosa* XQ-20044 suspension (approximately OD_540_ = 0.1) was fed to the *Poterioochromonas* sp. or *Hemiurosomoida* sp. cultures in a mixed ratio of 1:1 (v/v) every 2–3 days. By this way, successive transfer cultures of *Poterioochromonas* sp. and *Hemiurosomoida* sp. were established, which greatly facilitated the experiments.

### Control of *Poterioochromonas* sp. and *Hemiurosomoida* sp. using surfactants

Based on the successive transfer cultures of the two predators, the effects of SDBS, CDEA, SDS, AEO-7, and AES on *Poterioochromonas* sp. and *Hemiurosomoida* sp. were studied. Immediately after feeding with *Chlorella*, the cell densities of *Poterioochromonas* sp. and *Hemiurosomoida* sp. were counted and recorded. Generally, by feeding 1 L of the grazer culture with 1 L diluted *Chlorella* suspension every 2 days, the cell densities of *Poterioochromonas* sp. and *Hemiurosomoida* sp. reached at least 10^4^ cells mL^−1^ and 8 × 10^2^ cells mL^−1^, respectively, which ensured fast growth during the following days if no surfactant was added. These predator cultures were transferred into small flasks (80 mL working volume) and different volumes of the concentrated surfactant solutions were added to reach the desired concentrations. The flasks were kept at room temperature (25 °C) with low light and occasional shaking. The experiments were performed in triplicate. A control culture that without surfactant addition was included in each experiment to show predator viability and effectiveness of using the successive transfer culture. Morphological changes during the first hour were observed using an optical microscope and recorded with a digital camera. After 24 h of surfactant exposure, the cell densities of the live *Poterioochromonas* sp. and *Hemiurosomoida* sp. were counted.

### Field test of sodium dodecyl benzene sulfonate (SDBS) as a pesticide to control predatory flagellate and ciliate contaminations

SDBS was used as a pesticide to control *Poterioochromonas* sp. and *Hemiurosomoida* sp. in *Chlorella* mass cultivation. The field test was conducted at the microalgal mass culture test station at Yunnan province, China (26°29′29.6′′ N; 100°40′56.12′′ E). Detailed information about the raceway ponds and general cultivation parameters can be seen in our previous study [37]. *Chlorella pyrenoidosa* XQ-20044 was firstly cultivated in greenhouse-covered raceway ponds (5 m^2^, 1000 L). Then, approximately 2 L of the *Poterioochromonas* sp./*Hemiurosomoida* sp. culture suspension was added empirically into each pond on the 2nd or 3rd day. The grazer cultures acted as seeds to bring about *Poterioochromonas* sp./*Hemiurosomoida* sp. contamination, which was validated later by microbial community analysis using metagenomics data. 1 − 2 days after the addition, the predators increased in density and could be easily observed under the microscope and counted using a counting chamber. After several days of cultivation, the predator density had a marked increase, and then 10 mg L^−1^ SDBS was added to the ponds. For the control experiments, the development of the two predators was not interfered with by any extra operation. The experiments were conducted in parallel. Chl *a* content and predator density were monitored every day to indicate *Chlorella* growth and predator development, respectively.

The SDBS pesticide was also applied in a 20–200 m^2^ cascade culture of *Chlorella* in October 2019. The cultivations were performed according to our previous study [37] and continued for 20 days. For the first 10 days, the cultivation was conducted in a greenhouse-covered 20-m^2^ raceway pond (4000 L cultural volume) and then the culture suspension was transferred to a 200-m^2^ open raceway pond (40,000 L cultural volume) to inoculate a new cultivation. The cascade culture was microscopically monitored twice a day and two rounds of naturally occurring bio-contamination were observed. The SDBS pesticide (10 mg L^−1^) was used to control these contaminations.

### Measurements

Biomass dry weight (DW) and Chlorophyll *a* (Chl *a*) content were measured according to previous study [38] to evaluate *Chlorella* growth.. An equation (DW g L^−1^ = 38.14 × Chl *a* mg mL^−1^, *R*^2^ = 0.9979) was estimated from an uncontaminated parallel culture and used to calculate *Chlorella* DW for those samples that had contamination during the field test.

The photosynthetic status of *Chlorella* was evaluated by measuring the chlorophyll fluorescence parameter (*F*_V_/*F*_M_) using a PAM 2500 fluorometer according to Xie et al.’s method [39]. Fluorescein diacetate (FDA) staining was used to determine *Chlorella* viability (cell membrane integrity) according to the methodology described by Serra-Maia et al. [40]. Pictures were also taken on a Leica DMi8 C microsystem.

Cell densities of *Poterioochromonas* sp. and *Hemiurosomoida* sp. were counted with two types of plankton counting chamber (0.1 mL, 400 counting squares and 0.1 mL, 100 counting squares) after fixing with Lugol’s solution. Only 1 µL of Lugol’s solution (10%) was used for each 10 mL of sample to inhibit predator swimming but avoid cell disruption. At least three independent countings were conducted for each sample. As surfactants induced cell disintegration, all intact cells observed under microscope were recognized as live cell. To study the morphological changes of predator cells exposure to surfactant, the cells were continuously monitored under microscope, and a small device was used to assist video recording. Description of the device and the recorded videos can be seen in Additional file 3. The outdoor samples were also subjected to metagenomic sequencing to evaluate whether the microbial community was consistent with that expected.

All the above analytical experiments were performed in triplicate and the results were analyzed for variance using SAS 9.13 at a significance level of *α* = 0.05. Tukey’s multiple comparison tests were done where applicable.

## Supplementary Information

**Additional file 1.** Effects of surfactants (CDEA, SDS, AES, AEO-7) on growth of the alga *Chlorella pyrenoidosa* XQ-20044.

**Additional file 2.** Taxonomic classification of organisms in *Chlorella* cultures (5 m^2^ raceway pond) using metagenomics data at the 3rd day after inoculation with *Poterioochromonas* or *Hemiurosomoida*.

**Additional file 3.** Videos showing disintegration of *Poterioochromonas* and *Hemiurosomoida* cells exposure to SDBS pesticide (10 mg /L) and schematic diagram of the device used to assist video recording.

## Data Availability

All data generated or analyzed during this study are included in this published article and its supplementary information files.
