# Supplementary material for: Controlling of two destructive zooplanktonic predators in Chlorella mass culture with surfactants
Source: Biotechnol Biofuels. 2021 Jan 14;14:21. doi: 10.1186/s13068-021-01873-6 (PMC7809840; doi:10.1186/s13068-021-01873-6)
Supplement: Supplementary file 1 — Additional file 1. Effects of surfactants (CDEA, SDS, AES, AEO-7) on growth of the alga Chlorella pyrenoidosa XQ-20044. [file 13068_2021_1873_MOESM1_ESM.pptx]

## Slide 1
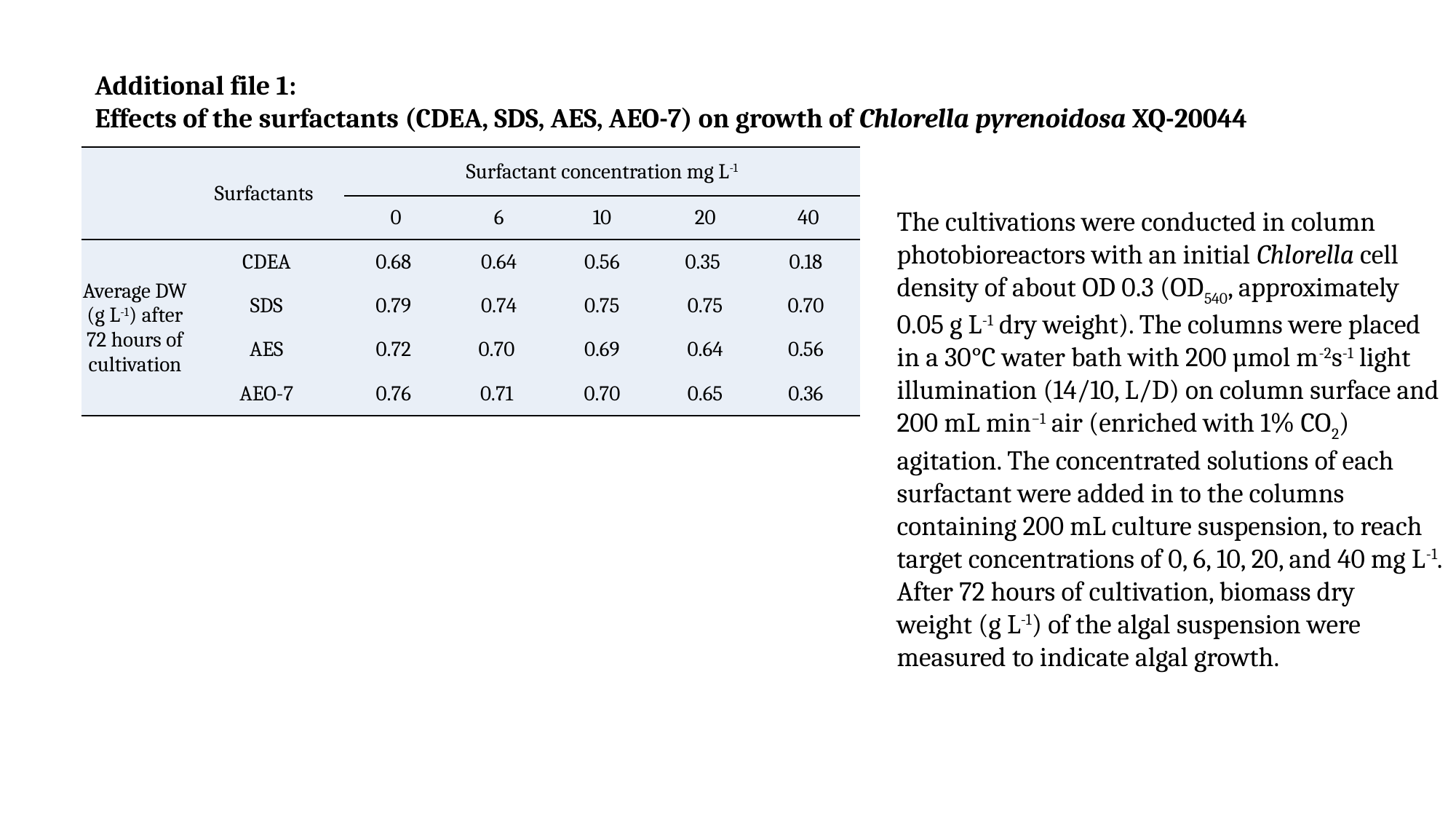

Additional file 1:
Effects of the surfactants (CDEA, SDS, AES, AEO-7) on growth of Chlorella pyrenoidosa XQ-20044
| | Surfactants | Surfactant concentration mg L-1 | | | | |
| --- | --- | --- | --- | --- | --- | --- |
| | | 0 | 6 | 10 | 20 | 40 |
| Average DW (g L-1) after 72 hours of cultivation | CDEA | 0.68 | 0.64 | 0.56 | 0.35 | 0.18 |
| | SDS | 0.79 | 0.74 | 0.75 | 0.75 | 0.70 |
| | AES | 0.72 | 0.70 | 0.69 | 0.64 | 0.56 |
| | AEO-7 | 0.76 | 0.71 | 0.70 | 0.65 | 0.36 |
The cultivations were conducted in column photobioreactors with an initial Chlorella cell density of about OD 0.3 (OD540, approximately 0.05 g L-1 dry weight). The columns were placed in a 30°C water bath with 200 µmol m-2s-1 light illumination (14/10, L/D) on column surface and 200 mL min−1 air (enriched with 1% CO2) agitation. The concentrated solutions of each surfactant were added in to the columns containing 200 mL culture suspension, to reach target concentrations of 0, 6, 10, 20, and 40 mg L-1. After 72 hours of cultivation, biomass dry
weight (g L-1) of the algal suspension were measured to indicate algal growth.
