# Supplementary material for: Controlling of two destructive zooplanktonic predators in Chlorella mass culture with surfactants
Source: Biotechnol Biofuels. 2021 Jan 14;14:21. doi: 10.1186/s13068-021-01873-6 (PMC7809840; doi:10.1186/s13068-021-01873-6)
Supplement: Supplementary file 2 — Additional file 2. Taxonomic classification of organisms in Chlorella cultures (5 m2 raceway pond) using metagenomics data at the 3rd day after inoculation with Poterioochromonas or Hemiurosomoida. [file 13068_2021_1873_MOESM2_ESM.pptx]

## Slide 1
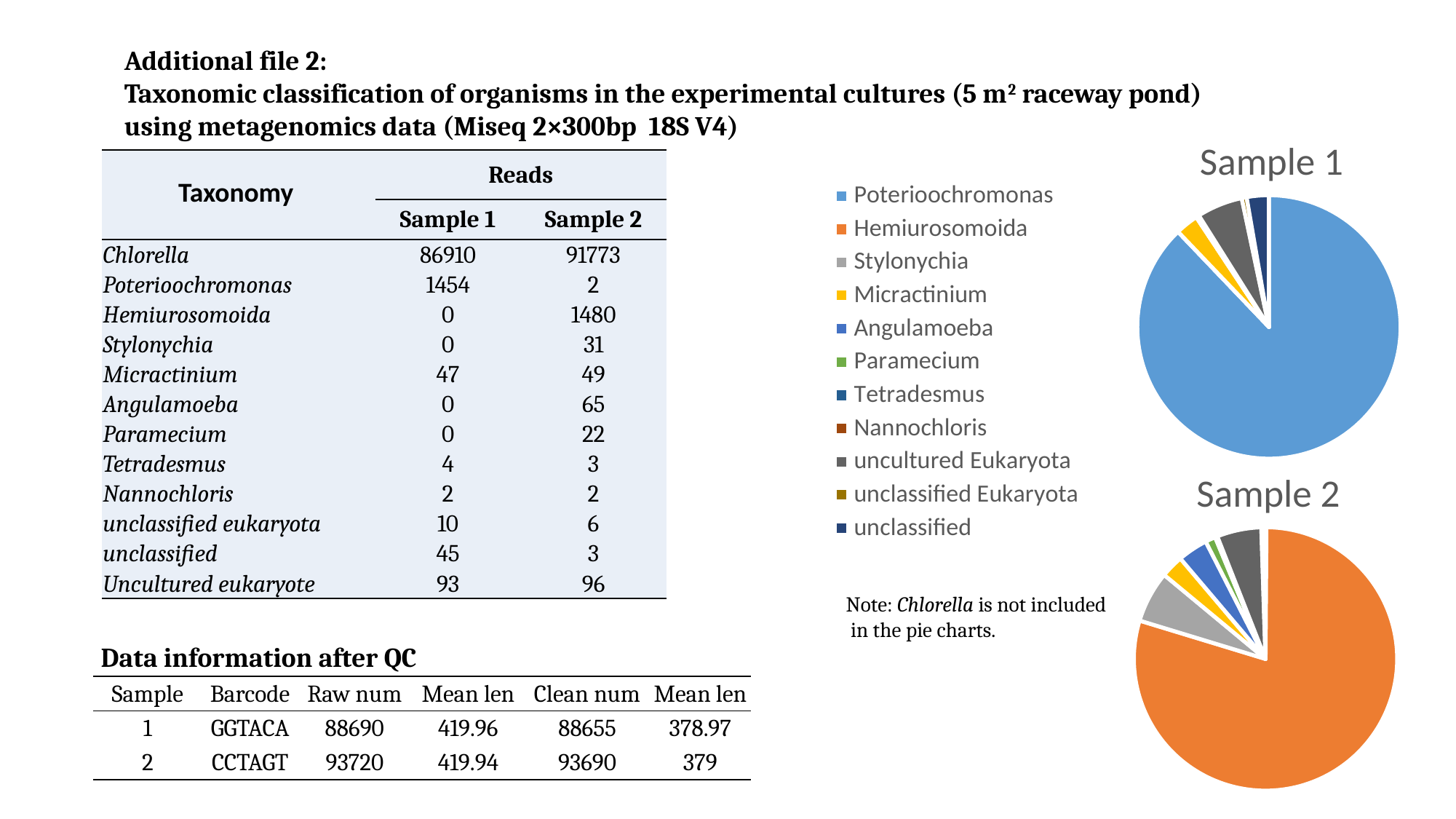

Additional file 2:
Taxonomic classification of organisms in the experimental cultures (5 m2 raceway pond)
using metagenomics data (Miseq 2×300bp 18S V4)
Note: Chlorella is not included
 in the pie charts.
| Taxonomy | Reads | |
| --- | --- | --- |
| | Sample 1 | Sample 2 |
| Chlorella | 86910 | 91773 |
| Poterioochromonas | 1454 | 2 |
| Hemiurosomoida | 0 | 1480 |
| Stylonychia | 0 | 31 |
| Micractinium | 47 | 49 |
| Angulamoeba | 0 | 65 |
| Paramecium | 0 | 22 |
| Tetradesmus | 4 | 3 |
| Nannochloris | 2 | 2 |
| unclassified eukaryota | 10 | 6 |
| unclassified | 45 | 3 |
| Uncultured eukaryote | 93 | 96 |
Data information after QC
| Sample | Barcode | Raw num | Mean len | Clean num | Mean len |
| --- | --- | --- | --- | --- | --- |
| 1 | GGTACA | 88690 | 419.96 | 88655 | 378.97 |
| 2 | CCTAGT | 93720 | 419.94 | 93690 | 379 |
