# Supplementary material for: Controlling of two destructive zooplanktonic predators in Chlorella mass culture with surfactants
Source: Biotechnol Biofuels. 2021 Jan 14;14:21. doi: 10.1186/s13068-021-01873-6 (PMC7809840; doi:10.1186/s13068-021-01873-6)
Supplement: Supplementary file 3 — Additional file 3. Videos showing disintegration of Poterioochromonas and Hemiurosomoida cells exposure to SDBS pesticide (10 mg /L) and schematic diagram of the device used to assist video recording. [file 13068_2021_1873_MOESM3_ESM.pptx]

## Slide 1
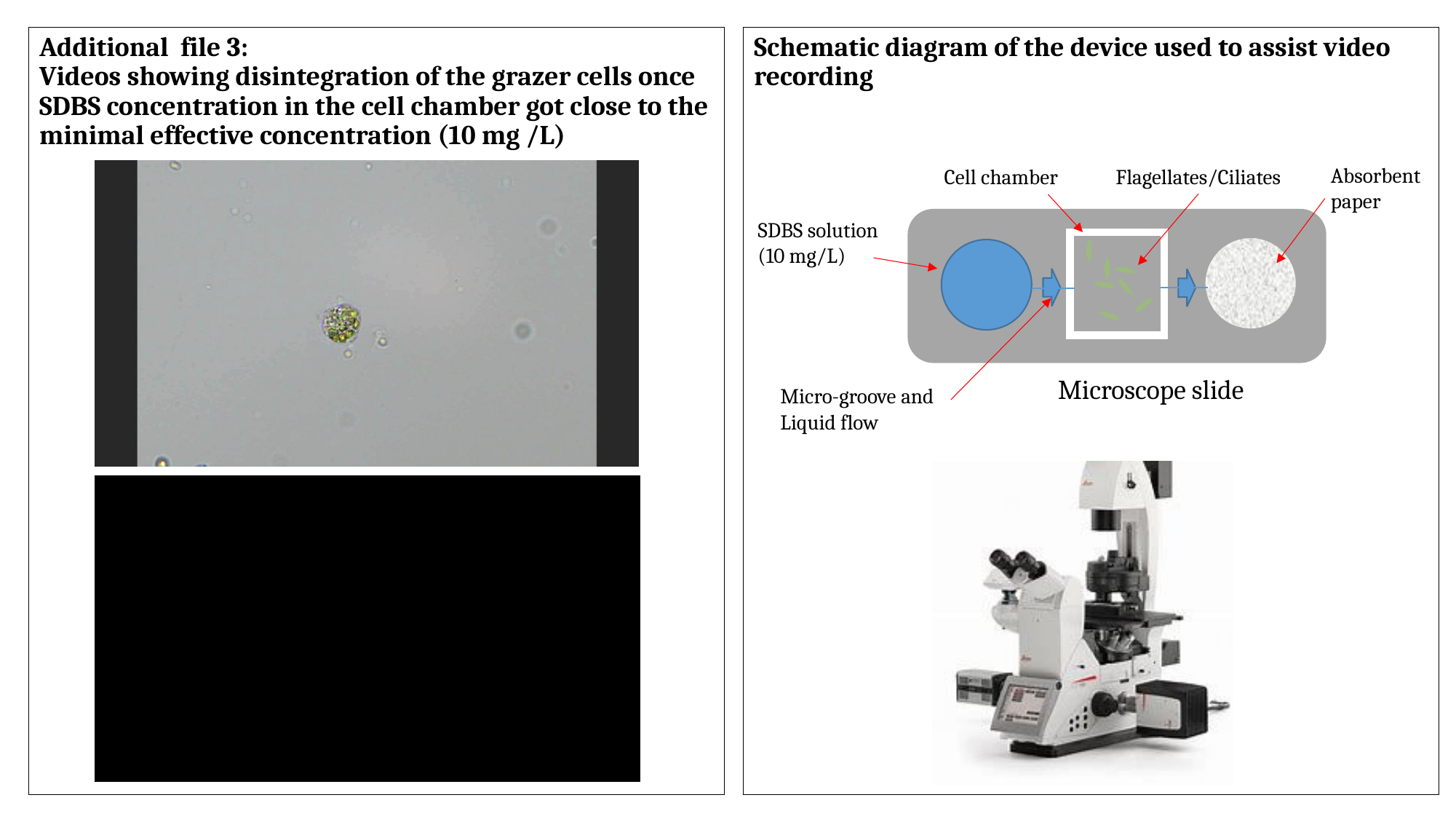

Additional file 3:
Videos showing disintegration of the grazer cells once SDBS concentration in the cell chamber got close to the minimal effective concentration (10 mg /L)
Schematic diagram of the device used to assist video recording
Absorbent
paper
SDBS solution
(10 mg/L)
Cell chamber
Flagellates/Ciliates
Microscope slide
Micro-groove and
Liquid flow
